# Which way to grow? Force over time may be the heart’s *Dao de jing*

**DOI:** 10.21542/gcsp.2016.21

**Published:** 2016-06-30

**Authors:** Pieter P. de Tombe, Peter Kohl

**Affiliations:** 1 Department of Cell and Molecular Physiology, Loyola University Chicago, Stritch School of Medicine, Maywood, IL 60304, USA; 2 Cardiac Biophysics and Systems Biology, National Heart and Lung Institute, Imperial College London, UK; 3 Institute for Experimental Cardiovascular Medicine, University Heart Centre Freiburg - Bad Krozingen, and Faculty of Medicine, Albert Ludwigs University Freiburg, Germany

## Background

Genetic cardiomyopathy manifests as either a hypertrophic or dilated phenotype. However, molecular mechanisms that determine which disease pathway emerges in patients is largely unknown. Work from the Molkentin laboratory published in the May issue of the journal Cell provides novel insights into this fundamental question. The investigators found that sarcomeric mutations associated with a reduced muscle contraction-time integral resulted in a dilated cardiomyopathy, while mutations associated with an increase in this parameter were associated with a hypertrophic phenotype. The molecular cellular cues that orchestrate which cardiomyopathic pathway ensues appear to be the signal transduction pathways involving the molecules MEK1 and ERK1/2. The identified signals driving overall growth of the heart, on the either hand, were found to involve Calcineurin and NFAT. These findings may help improve treatment strategies aimed to combat familial cardiopathy and, moreover, pave the way to the development of novel personalized medicine based therapy by using cardiac cells that are derived from individual patient’s induced pluripotent stem (iPS) cells.

## Introduction

Cardiac hypertrophy may manifest as either (*i*) concentric, where the walls of the heart thicken and cavity volume is reduced, or (*ii*) eccentric, where wall thickness is either maintained or even reduced while cavity volume is enlarged. In both cases, overall ventricular mass is enlarged and, thus, both pathological conditions qualify as cardiac hypertrophy. Apart from obvious geometric differences, cardiac pump function is also affected quite differently: a concentric hypertrophied heart tends to perform at normal or even supra-normal strength during systole while filling in diastole may be impaired, particularly at higher heart rates. In contrast, the eccentric hypertrophied heart tends to suffer from weakened systolic pressure generation while diastolic filling is either normal or impaired. Cardiac hypertrophy can be acquired or genetically determined^1^.

Acquired concentric cardiac hypertrophy may develop when an elevated after-load (pressure against which ejection occurs) is placed upon the heart, be it by an obstructed outflow valve or by systemic hypertension (“pressure overload”). Acquired eccentric hypertrophy, in contrast, may develop upon pathologically increased pre-load (end-diastolic volume), for example when the ventricular outflow valves are leaky (“volume overload”). Both conditions are associated with inefficient cardiac pump function.

Another etiology leading to acquired eccentric cardiac hypertrophy is late stage ischemic heart disease caused by, for example, coronary atherosclerosis. A single large myocardial infarction, or more often a series of smaller myocardial infarcts, severely impairs cardiac pumping, which ultimately sets the stage for a remodeling process that ends with a dilated and enlarged (hypertrophied) heart; the mechanisms underlying the transition into dilated eccentric hypertrophied heart are not very well understood.

Concentric hypertrophy is often induced experimentally in animal models by mechanical loading of the left ventricle by artificial aortic outflow obstruction, a procedure called *transverse aortic constriction* (or TAC). In the widely employed mouse TAC model, the initial concentric hypertrophy and hyper-contractility is often followed in time by cardiac dilation and development of a pronounced eccentric cardiac hypertrophy and hypo-contractility. Whether and how frequently such a shift occurs in large animals and, importantly, in humans is not entirely clear at present.

Genetic cardiac hypertrophy is quite different and is, as the name suggests, caused by mutations that predispose an individual to “spontaneously” develop concentric or eccentric cardiac hypertrophy. Familial hypertrophic cardiomyopathy (FHC) is a syndrome where patients present with concentric hypertrophy that cannot be explained on the basis of hemodynamic loading conditions such as described above. Hypertrophy of the upper septum close to the ventricular outflow tract is often observed, which can be a source of profoundly increased resistance to blood flow during ventricular ejection that results in markedly elevated end-systolic ventricular pressure, in particular during exercise. Ventricular arrhythmias are common and, sometimes, sudden death is the first (and last!) manifestation of underlying FHC in young athletes that have been asymptomatic and undiagnosed. The identification of other afflicted family members presenting with various degrees of unexplained concentric cardiac hypertrophy solidifies the FHC diagnosis.

A major leap forward occurred a quarter century ago when the Seidman group published the discovery of the first association between FHC and a mutation in the sarcomeric protein myosin (R403Q), a phenotype that could be recapitulated in a genetic mouse model^2^. Since that pioneering work, over 1,500 different mutations have been identified that associate with FHC. Most, but not all, mutations have been found in sarcomeric proteins, prompting classification of FHC as a “disease of the sarcomere”. The majority of FHC mutations are found in the two proteins: the motor protein myosin (MHC) and the thick-filament accessory protein myosin binding protein C (MyoBPC). The incidence of FHC-causing mutations in the general population is thought to be ∼1:500, though recent reports suggest an even higher incidence of 1:200^3^. One puzzling aspect of human FHC mutations is the variable penetrance, where similarly afflicted mutation carriers within a family display vastly different phenotypes that vary from early onset and pronounced concentric cardiac hypertrophy, ventricular outflow obstruction, or predisposition to ventricular arrhythmias at one end of the spectrum, to essentially phenotype-negative family members well into adulthood at the other extreme^4^. This is in contrast to experimental animal models, where recapitulation of the very same mutation in murine lines often leads to a consistent display of the FHC phenotype. The variable penetrance in human not only underscores our limited understanding of the intricate relationships between genomic mutations and the FHC phenotype. It also greatly complicates the clinical management of FHC mutation-positive but asymptomatic and often young patients. Questions such as prophylactic implantation of an automatic defibrillator, limits on participation in certain sports activities either at school or during after-school events, genetic counseling regarding life decisions and family planning, etc., are very difficult to answer. An improved understanding of genomephenotype relations, with the ultimate goal of improving our ability to predict timing and progression of the human FHC syndrome phenotype is clearly needed to improve health care delivery for these individuals^5^.

Until recently, the notion that genetic eccentric cardiac hypertrophy exists was called into question. Certainly, a sizable proportion of patients (up to ∼30%) present with unexplained enlarged and dilated hearts, a syndrome classified as idiopathic dilated cardiomyopathy, as opposed to the acquired end-stage dilated ischemic cardiomyopathy described above^1^. However, investigators speculated that a significant portion of these so-called ‘idiopathic’ cases were immunologically derived, either in response to unrecognized/undiagnosed infections or auto-immunological responses. None withstanding, the notion was challenged by publications, albeit infrequent, reporting sarcomeric as well as non-sarcomeric mutations that associate with dilated cardiomyopathy/eccentric cardiac hypertrophy.

The consensus changed dramatically in 2012 when the Seidman group reported a strong association between truncation mutations in the titin gene and non-acquired human dilated cardiomyopathy^6^. Titin, the largest protein known to date, is derived from a single gene, forms an important elastic and signaling component of the contractile apparatus^7^, and is referred to as ‘the third filament’ of the sarcomere. Recent studies show that titin mutations are the most common cause of human genetically-caused dilated cardiomyopathy^8^, confirmed the impact of titin mutations on contractile biology in-vitro^9^, and even suggested a role for titin mutations in peri-partum cardiomyopathy^10^. Thus, the emerging consensus is that both concentric and eccentric cardiac hypertrophy indeed can be of genetic origin, and mutations appear to underlie the majority of FHC and familiar dilated (FDCM) cardiomyopathies^1^.

So what determines whether a particular mechanical loading condition or mutation leads to concentric or eccentric cardiac hypertrophy? This is a fundamental question that, when ultimately answered, may also shed light on why the relation between a genetic mutation and the development of a phenotype in humans is so variable and unpredictable. At the cellular level, concentric cardiac hypertrophy involves myocytes that are of normal length but increased cross-sectional area, which is accomplished by in-parallel replication of the contractile apparatus units (the sarcomeres). Conversely, in eccentric cardiac hypertrophy cells are elongated with normal cross-sectional area and this is accomplished by replication of sarcomeres in-series. The former allows increased peak force generation by a cell, the latter supports larger net changes in cell length. This principal difference makes it difficult to compare remodeling of cell contractility side-by-side, as measurements of shortening may over-report the changes seen in eccentric cardiac hypertrophy (in particular in mechanically unloaded cells whose sarcomere lengths are below *in situ* levels^11^), while measurements of intact cell force (difficult, but possible^12^) may be more sensitive to changes in concentric cardiac hypertrophy. In any case – without being able to control pre- and afterload, single cell comparisons dominated by in-series versus in-parallel addition of sarcomeres is bound to be difficult^13^.

The cellular regulatory processes responsible for the divergent hypertrophic remodeling of cardiac myocytes are largely unknown. Yet, remarkably in case of acquired cardiac hypertrophies, the observed cellular responses are precisely matched to the type of abnormal mechanical load the heart is facing: the need for a stronger pump in case of “pressure overload”, and for a larger stroke volume in case of “volume overload”. However, it is not entirely clear how one would resolve the ‘chicken-and-egg’ dilemma of exploring what came first.

In genetic FHC or FDCM, the cellular signals that lead to either a concentric or eccentric cardiac hypertrophic heart are equally unclear. It has been suggested that mutations that *increase* the responsiveness of the cardiac myofilaments to calcium ions lead to FHC while, conversely, mutations that *reduce* myofilament calcium sensitivity result in FDCM^14,15^. This notion is based largely on experimental animal research, either by use of recombinant proteins or genetically modified murine models, where equivalents to the human mutations were found to either increase or decrease myofilament calcium sensitivity. Likewise, in cases were human cardiac samples are available (mostly from septal myectomy of ventricular outflow tract obstructions in FHC), an increase in myofilament calcium sensitivity is frequently seen. The “myofilament calcium sensitivity” hypothesis was recently put to test in an elegant series of experiments by the Molkentin lab, reported in the May 2016 issue of the journal *Cell*^16^.

**Figure 1.**
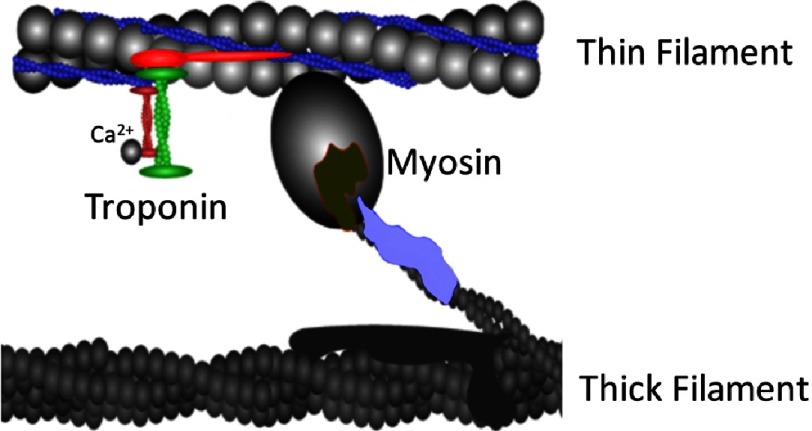
Schematic illustration of the structure of the cardiac sarcomere. Contractile activity of the cardiac myofilaments derives from the interaction between actin (located in the thin filament) and myosin (located in the thick filament). Contraction commences when Ca^2+^ ions bind to Troponin-C (dark red), the Ca^2+^ receptor of the trimeric troponin complex that also contains Troponin-I (green; an inhibitor of the actin-myosin interaction in the absence of Ca^2+^ binding), and Troponin-T (bright red; anchors the troponin complex to thin filament). The latter communicates with tropomyosin (dark blue filaments located in the grooves of the actin coiled-coil helical filament). Tropomyosin movement upon TnC Ca^2+^ binding uncovers the myosin binding site on actin to initiate contraction. Myosin is an asymmetric protein containing a globular head domain that can bind to actin and undergo structural conformational changes upon hydrolysis of ATP that results in contractile force development and shortening; the myosin tail domain intertwines with other myosin tails to form the back-bone of the thick filament. Two regulatory light chains (regulatory light chain, dark green; essential light chain, light blue) are shown located within the globular head domain of myosin; these proteins modulate contractile activity. Myosin binding protein C, a protein who’s function is not fully understood, is shown in dark grey as a component of the thick filament. Over 1,500 mutations in sarcomeric proteins have been identified to date, which are associated with familial hypertrophic or dilated cardiomyopathy.

**Figure 2.**
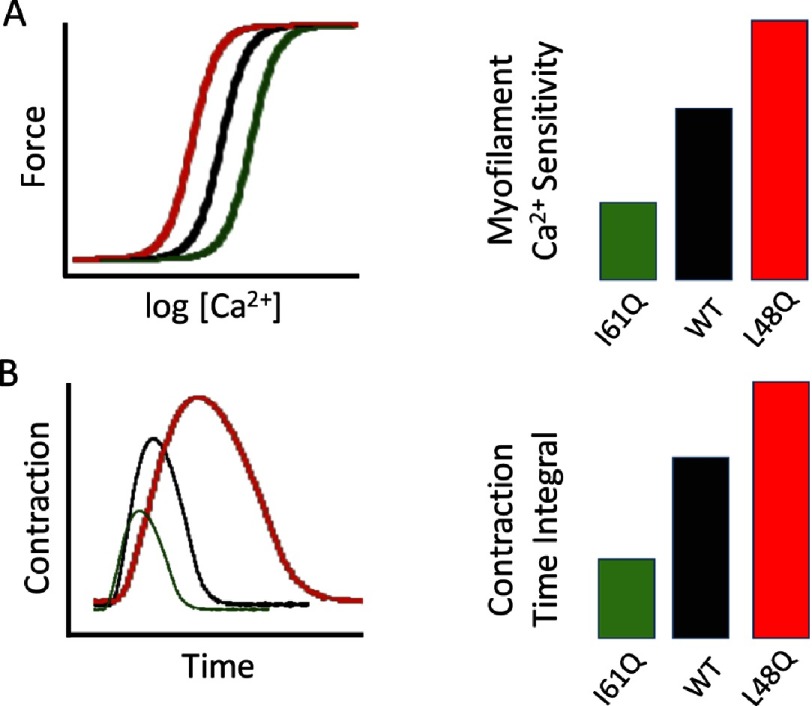
Troponin C mutations that induce altered myofilament calcium sensitivity. The impact of two separate mutations in Troponin-C (TnC) are schematically illustrated. Panel A: TnC-I61Q (green) results in decreased myofilament calcium sensitivity, while TnC-L48Q (red) leads to increased calcium sensitivity; all compared to wildtype (WT) TnC shown in black. This is reflected in a left- (L48Q) or a right-shift (I61Q) in the relationship between activator Ca^2+^ and myofilament force development (left), as well as myofilament Ca^2+^ sensitivity as indexed by the mid-point of these force-Ca^2+^ relationships (right). Panel B: changes in myofilament calcium sensitivity are expected to alter peak myocardial twitch force as well as the overall duration of the contractile event (left panel). The combined effects lead to an altered contraction-time integral index (the area under the curve of the contraction waveform) as shown in the right panel.

Rather than recapitulating a specific genetic cardiomyopathy mutation in murine models, these investigators started out by introducing one of two “mutations” in the gene that encodes the contractile protein Troponin-C (TnC). TnC is the calcium receptor protein of the troponin regulatory complex found in striated muscle (Figure 1), which regulates the initiation of muscle contraction in response to the action potential induced calcium transient that causes cardiomyocytes to contract^17^. The mutations in question were not “real mutations”, in the sense that the amino acid substitutions that were induced in TnC are not associated with any known disease. Instead, these mutations were previously reported to cause either an increase (L48Q) or a decrease (I61Q) in calcium binding affinity of TnC and, more importantly, a change in myofilament calcium sensitivity when the mutated TnC proteins are incorporated into the cardiac sarcomere. The impact of these mutations of myofilament calcium sensitivity and the resulting alterations in contractile function is schematically illustrated in Figure 2. Thus, the investigators asked the crucial question: if myofilament calcium sensitivity is increased in concentric hypertrophy and decreased in eccentric hypertrophy, is the opposite also true. That is, does increased or decreased myofilament calcium sensitivity *lead* to hypertrophic or dilated cardiomyopathy (respectively)?

The short answer Davis et al. report is: yes!

Experimental mice in whom TnC is more sensitivity to calcium were prone to develop concentric cardiac hypertrophy, particularly when heart rate and/or cardiac contractility was blunted, while eccentric cardiac hypertrophy was found to develop when TnC calcium sensitivity was experimentally reduced.

Interestingly, eccentric cardiac hypertrophy could be prevented by cross-breeding the I61Q mouse with the myosin R403Q FHC model, which is known to be associated with increased myofilament calcium sensitivity. This makes much ‘sense’, in as far as a reduction in contractility will make it more difficult for the heart to respond to the kinds of transient increases in end-diastolic load that accompany normal physical activity. Dilation, once started, then can become self-perpetuating in view of the inverse relation between chamber radius and pressure generating ability of cardiomyocytes, highlighted by the Law of Laplace.

**Figure 3.**
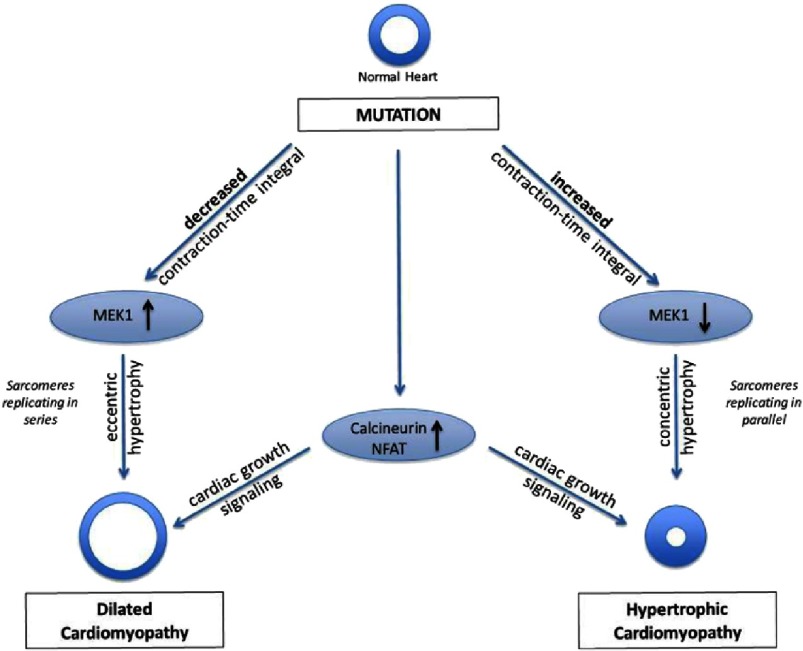
Genetic, as opposed to acquired, cardiac hypertrophy is now known to be caused, in many cases, by mutations. What was not known, however, what cellular and molecular mechanisms determine whether a mutation leads to Familiar Dilated Cardiomyopathy (FDCM; left) or Familiar Hypertrophic Cardiomyopathy (FHC; right). Work by Davis et al.^16^ in the May 2016 issue of the Journal *Cell* has provided important new insights into this important question. The contraction-time-integral, determined on cardiac myocytes in isolation, may be a powerful predictor of the type of cardiac hypertrophy that is found *in vivo*. Moreover, the activity of the signaling protein *mitogen-activated protein kinase kinase 1* (MEK1), the upstream regulator of *extracellular signal-related kinase 1/2* (ERK 1/2), may constitute the molecular mechanism by which the mechanical signal is translated to cue cells to either replicate sarcomeres in series (left) or in parallel (right) to the existing contractile apparatus. On the other hand, the *calcium responsive serine-threonine phosphatase Calcineurin* and, consequently, its downstream *transcriptional effector of activated T cells* (NFAT) may be central to the regulation of overall cardiac hypertrophy.

To gain insight into what cellular mechanisms may be involved, the investigators next embarked on an extensive set of experiments involving both Western-blot probing of molecules known to be central in cardiac hypertrophy signaling as well as cross-breeding the TnC mutation mice with numerous mouse models in which the activity of these very same signaling proteins is either blunted or enhanced. The combined result of these experiments is the finding that increased myofilament calcium sensitivity and the resultant eccentric dilated cardiac hypertrophy was associated, and potentially even induced by decreased activity of the signaling protein *mitogen-activated protein kinase kinase 1* (MEK1), the upstream regulator of *extracellular signal-related kinase 1/2* (ERK 1/2), which is known to be intimately involved in cardiac hypertrophic cell signaling. Conversely, concentric hypertrophic cardiac hypertrophy may be caused by an increase in MEK1 activity (see Figure 3).

On the other hand, the *calcium responsive serine-threonine phosphatase* Calcineurin and, consequently, its downstream *transcriptional effector of activated T cells* (NFAT), important regulators of cardiac hypertrophy, were upregulated in both forms of cardiac hypertrophy induced by the TnC mutations.

What cellular clues may drive these signals? Davis et al. examined cell shortening and calcium transients in myocytes isolated from the hearts of the L48Q and I61Q TnC mutant mice, as well as data from previously published reports on isolated myocyte data from FDCM (Δ210 troponin T) and restrictive cardiomyopathy (R193H TnI) mouse models. Similar data were obtained from cardiac myocytes derived from human *inducible pluripotent stem cells* (IPS) that harbored mutations associated with FHC (either myosin or myosin binding protein C) or FDCM (either troponin T or phospholamban). Both human and murine myocytes harboring mutations associated with hypertrophic cardiomyopathies showed increases in the integral of mechanical activity over time. To describe this, the *area under the curve* of mechanical myocyte activity was quantified (contraction-time-integral; CTI). Conversely, cells harboring mutations associated with FDCM showed a reduction in CTI. When combined into a statistical model, a continuous and proportional relationship was found between the extent of concentric or eccentric cardiac hypertrophy and the integral of force development over time.

The overall interpretation of these results is that MEK1 signaling directs the hypertrophic program towards the appropriate cardiac geometry, and this is associated with changes in cellular mechanical activity, reflected by the CTI parameter, while Calcineurin/NFAT signaling directs overall cardiac hypertrophy (growth). These ideas are schematically illustrated in Figure 3.

It should be noted that Davis et al. did not measure cell contractile force directly. Instead, sarcomere shortening and calcium transients were experimentally assessed in isolated cardiac myocytes, and these data were then used to calculate force using a mathematical model. The authors were careful, though, to refer to “area under the tension/shortening curve” in their presentation.

The notion that the single unifying CTI parameter, which may be approximated by simply monitoring shortening of isolated myocyte, is a potentially powerful descriptor of cardiac hypertrophy may open the possibility of personalized medicine. That is, cardiomyocytes could be prepared from IPS cells derived from tissue samples of individual patients who are positive for FDCM or HCM associated mutations with the goal to assess the mechanical contraction integral. This parameter could possibly then be used to predict the patient’s potential future disease severity and its progression. It may also serve to solve the long standing puzzle regarding the variable penetrance that is often observed for these mutations. Another application of the IPS/CTI approach would be individual patient examination of existing approved or experimental pharmaceuticals, that is, screening for individual effectiveness. At present, treatment strategies for FHC or FDCM patients are limited and largely based on experience gained from acquired cardiac hypertrophies. These therapies may not be appropriate for genetic versions of the syndromes. Fortunately, much is already known about the molecular mechanisms underlying striated muscle contraction^17^, and some agents have been identified that directly target myofilaments to cause reduced calcium sensitivity such as the polyphenols cathechins found in green tea^18^ or the β1 receptor-blocker nebivovol^19^ which could be helpful in the treatment of FHC. In turn, myosin *activators*, such as omecamtiv mecarbil^20^ currently in clinical trial, may prove useful as treatment strategy in FDCM.

**Figure 4.**
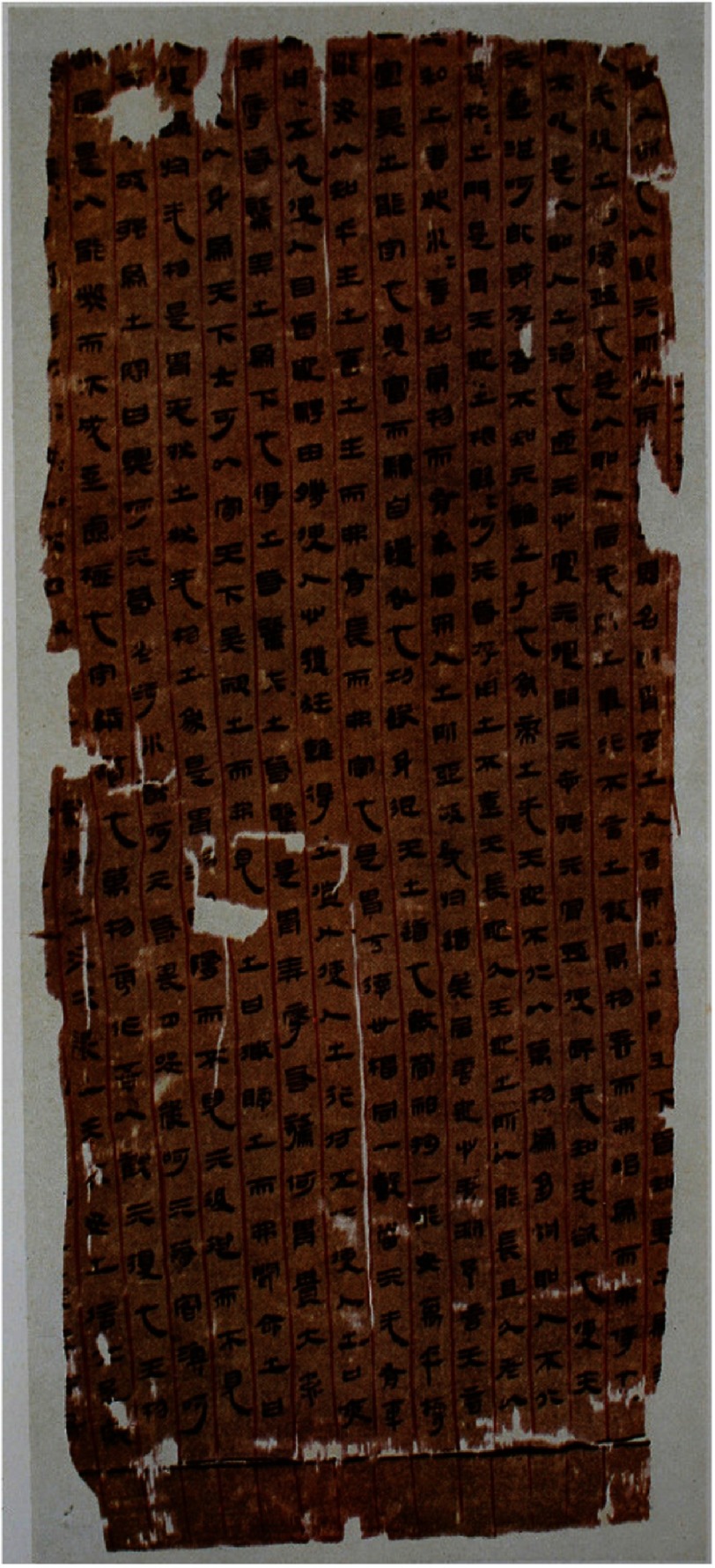
Part of a Taoist manuscript by an unknown author. Ink on silk, 2th century BCE, Han Dynasty, unearthed from Mawangdui tomb 3rd, Chansha, Hunan Province, China. Hunan Province Museum (reproduced from [21]).

Thus, the exciting work by Davis et al.^16^ is likely to provide a stimulus for further study of the molecular mechanisms underlying not only genetic, but also acquired cardiac hypertrophy and failure. For example, it will be important to establish whether the CTI parameter is truly unifying in being able to predict in all cases disease severity, progression, and responsiveness to treatment strategies. As such, *force over time*, may constitute the *Dao de jing* of the heart. Dao de jing, or Tao Te Ching, is an ancient Chinese classic text from ∼500 BC (Figure 4) that forms a foundation for philosophical and religious Taoism^21^. It is used as a source of inspiration for both artist and philosophers. The text is ascribed to Laozi, although his historical existence is a matter of scholastic debate. ‘Dao’ means “way”, ‘de’ may mean “virtue” or “divine power”, and ‘jing’ means “classic”. Hence one interpretation of Dao de jing is “the True Classic of the Power and the Way” – just as *force over time*. So perhaps the CTI approach may guide us towards the right pathways that lead to prediction and prevention of both concentric and eccentric hypertrophy.
